# The Effects of a Novel Hormonal Breast Cancer Therapy, Endoxifen, on the Mouse Skeleton

**DOI:** 10.1371/journal.pone.0098219

**Published:** 2014-05-22

**Authors:** Anne Gingery, Malayannan Subramaniam, Kevin S. Pitel, Jordan M. Reese, Muzaffer Cicek, Laurence B. Lindenmaier, James N. Ingle, Matthew P. Goetz, Russell T. Turner, Urszula T. Iwaniec, Thomas C. Spelsberg, John R. Hawse

**Affiliations:** 1 Department of Biochemistry and Molecular Biology, Mayo Clinic, Rochester, Minnesota, United States of America; 2 Department of Molecular Pharmacology and Experimental Therapeutics, Mayo Clinic, Rochester, Minnesota, United States of America; 3 Skeletal Biology Laboratory, School of Biological and Population Health Sciences, Oregon State University, Corvallis, Oregon, United States of America; 4 Department of Oncology, Mayo Clinic, Rochester, Minnesota, United States of America; INSERM U1059/LBTO, Université Jean Monnet, France

## Abstract

Endoxifen has recently been identified as the predominant active metabolite of tamoxifen and is currently being developed as a novel hormonal therapy for the treatment of endocrine sensitive breast cancer. Based on past studies in breast cancer cells and model systems, endoxifen classically functions as an anti-estrogenic compound. Since estrogen and estrogen receptors play critical roles in mediating bone homeostasis, and endoxifen is currently being implemented as a novel breast cancer therapy, we sought to comprehensively characterize the *in vivo* effects of endoxifen on the mouse skeleton. Two month old ovariectomized C57BL/6 mice were treated with vehicle or 50 mg/kg/day endoxifen hydrochloride via oral gavage for 45 days. Animals were analyzed by dual-energy x-ray absorptiometry, peripheral quantitative computed tomography, micro-computed tomography and histomorphometry. Serum from control and endoxifen treated mice was evaluated for bone resorption and bone formation markers. Gene expression changes were monitored in osteoblasts, osteoclasts and the cortical shells of long bones from endoxifen treated mice and in a human fetal osteoblast cell line. Endoxifen treatment led to significantly higher bone mineral density and bone mineral content throughout the skeleton relative to control animals. Endoxifen treatment also resulted in increased numbers of osteoblasts and osteoclasts per tissue area, which was corroborated by increased serum levels of bone formation and resorption markers. Finally, endoxifen induced the expression of osteoblast, osteoclast and osteocyte marker genes. These studies are the first to examine the *in vivo* and *in vitro* impacts of endoxifen on bone and our results demonstrate that endoxifen increases cancellous as well as cortical bone mass in ovariectomized mice, effects that may have implications for postmenopausal breast cancer patients.

## Introduction

It is estimated that nearly 1.5 million women will be diagnosed with breast cancer this year and nearly 70% of these individuals will have estrogen receptor alpha (ERα) positive tumors. Women with ERα positive tumors are treated with drugs that either suppress the production of estrogen (aromatase inhibitors [AIs]), or block estrogen signaling (e.g. tamoxifen), in order to reduce the risk of breast cancer recurrence. At present, the drugs of choice for postmenopausal women are typically AIs as they have been shown to have equal or improved benefit relative to tamoxifen [1], [2], [3], [4]. While AIs have proven to be a successful class of breast cancer drugs, a major side effect of such therapies is accelerated bone loss and increased rates of vertebral and hip fractures [5], [6]. These negative skeletal effects result from blockade of aromatase, the enzyme which converts androgens to estrogens and is the primary source of estrogen in postmenopausal women. Many postmenopausal breast cancer patients already have evidence of bone loss and are at increased risk for fracture, therefore, maintenance of bone mass in breast cancer patients is critical as additional loss of bone in these individuals leads to loss of height, severe back pain, permanent disability and even death should hip or serious vertebral fractures occur. These issues led to the convening of an American Society of Clinical Oncology Task Force which concluded that “oncology professionals, especially medical oncologists, need to take an expanded role in routine and regular assessment of their patients' bone health” [7].

To address the negative skeletal effects incurred by AIs, clinicians encourage the use of vitamin D and calcium and additionally may prescribe a class of drugs known as bisphosphonates [8], [9]. Bisphosphonates have become the drugs of choice for treating fractures and bone loss in postmenopausal women with osteoporosis [10], [11] as well as preventing cancer therapy induced bone loss in breast cancer patients treated with AIs [12], [13], [14]. Bisphosphonates function by inhibiting osteoclast-mediated bone resorption [15], [16] but they do not promote new bone formation. Therefore, the identification of highly effective breast cancer therapies that do not negatively impact the skeleton, or that actually exhibit beneficial effects on bone health, continue to represent a critical clinical need.

Selective estrogen receptor modulators (SERMs) have provided major therapeutic advances in addressing these issues since they exert both estrogen and anti-estrogen-like actions in a tissue dependent manner [17]. Compounds such as tamoxifen, raloxifene, lasofoxifene and arzoxifene have been shown to reduce bone loss and reduce the risk of fractures [18], [19], [20], [21], [22], [23]. Of these, raloxifene is currently the only FDA approved SERM for treating osteoporosis and reducing the risk of breast cancer [24], [25]. However, tamoxifen remains the most available and effective SERM for the prevention and treatment of breast cancer and has received approval for multiple breast carcinoma indications that cover the full spectrum of this disease.

Like many drugs, tamoxifen is a parent compound that undergoes significant metabolism in the human body. While 4-hydroxytamoxifen (4HT) is the most commonly studied metabolite, it represents less than 10% of tamoxifen primary oxidation [26], [27]. Recent data suggests that another hydroxylated metabolite, 4-hydroxy-N-desmethyl-tamoxifen (endoxifen), might be responsible for the majority of tamoxifen activity in the human body [28], [29], [30], [31], [32], [33], [34], [35], [36], [37], [38], [39], [40]. Studies in our laboratory have demonstrated that endoxifen, at the concentrations observed in the clinic, is the most active and potent tamoxifen metabolite with regard to its anti-breast cancer properties [41], [42]. Furthermore, we have provided evidence that endoxifen elicits differential gene expression profiles and activates unique biological pathways when compared to tamoxifen and its other metabolites [43].

Based on these preclinical data, and the clinical data demonstrating an association between CYP2D6 metabolism and endoxifen concentrations with breast cancer recurrence, a novel formulation (endoxifen hydrochloride) was synthesized and phase I clinical trials designed to evaluate the pharmacokinetics and safety of endoxifen in hormone responsive cancers that are refractory to standard therapies are under way at the Mayo Clinic (NCT ID: NCT01327781) and National Cancer Institute (NCT ID: NCT01273168). Phase II studies of endoxifen in AI refractory breast cancer will commence in 2014.

Based on the unique mechanism of action of endoxifen in breast cancer cells, its development and implementation as a novel breast cancer therapy and the importance of maintaining healthy bone in cancer patients, we sought to comprehensively characterize the *in vivo* effects of endoxifen on the mouse skeleton. The potential effects of endoxifen on the skeleton have never been reported and such effects cannot be extrapolated from past studies examining the actions of tamoxifen on bone due to differences in the metabolism of tamoxifen in rodents [44], [45]. Here, we detail the effects of endoxifen in an ovariectomized mouse model system through the use of dual-energy x-ray absorptiometry (DXA), peripheral quantitative computed tomography (pQCT), micro-computed tomography (micro-CT) and histomorphometric analyses. Additionally, we examine alterations in biochemical markers of bone formation and turnover and the *in vivo* impact of endoxifen treatment on bone forming osteoblast and bone resorbing osteoclast precursor cells as well as osteocytes. Our data provide evidence that endoxifen alters the activities of osteoblasts, osteoclasts and osteocytes resulting in compartment-specific alterations in bone mass and architecture relative to vehicle treated mice.

## Materials and Methods

### Animals, reagents and study design

For this study, we utilized 20 two-month-old female C57BL/6 wild-type mice. All mice underwent ovariectomy to induce gonadal hormone insufficiency and were subsequently randomized to two groups of 10 animals each which were treated with either vehicle control (0.375 mg/mL ascorbic acid) or endoxifen-hydrochloride (50 mg/kg/day) via oral gavage. Purified z-endoxifen hydrochloride was synthesized by the National Cancer Institute and obtained through the Developmental Therapeutics Program (NSC 750393). Fresh vehicle and endoxifen-hydrochloride solutions were prepared weekly and stored in the dark at 4°C. Treatments persisted daily for a total of 45 days. During this time, all mice were housed in a temperature controlled room (22±2°C) with a light/dark cycle of 12 hours. Animals had free access to water and were fed standard laboratory chow ad libitum.

### Ethics statement

This study was carried out in strict accordance with the recommendations in the Guide for the Care and Use of Laboratory Animals of the National Institutes of Health. The Mayo Clinic Institutional Animal Care and Use Committee (IACUC) approved all animal care and experimental procedures described in this paper (Protocol A22112). Bones and tissues were harvested after euthanasia following Mayo Clinic IACUC-approved CO_2_ inhalation protocols and all efforts were made to minimize suffering.

### Dual-energy x-ray absorptiometry (DXA) and peripheral quantitative computed tomography (pQCT)

Following 45 days of treatment, all mice underwent DXA and pQCT scans. For DXA analysis, a Lunar PIXImus densitometer (software version 2.10) was calibrated using a hydroxyapatite phantom provided by the manufacturer (Lunar Corp, Madison, WI). Subsequently, mice were anesthetized and whole body scans were conducted. Data analysis consisted of whole body lean mass, fat mass, bone mineral density and bone mineral content. For pQCT, mice were subsequently placed in a supine position on a gantry using the Stratec XCT Research SA+, software version 6.20C, (Stratec Medizintechnik Gmbh, Pforzheim, Germany). Slice images were measured at 1.9 mm (corresponding to the proximal tibial metaphysis) and 9 mm (corresponding to the tibial diaphysis) from the proximal end of the tibia to obtain trabecular and cortical parameters respectively as described previously [46].

### Micro-CT

Immediately following DXA and pQCT scans, mice were sacrificed using CO_2_ and left femora and tibiae, as well as 5^th^ lumbar vertebrae (LV5), were removed and fixed in 10% neutral buffered formalin overnight. Formalin fixed bones were transferred to 70% ethanol and stored at 4°C until time of analysis. Micro-CT was used for nondestructive three-dimensional evaluation of bone volume and cortical and cancellous bone architecture. Femora, tibiae and LV5 were scanned in 70% ethanol at a voxel size of 12×12×12 µm using a Scanco Micro-CT40 scanner (Scanco Medical AG, Brüttisellen, Switzerland) set at 55 kVp. The threshold for analysis was determined empirically and set at 245 (0-1,000 range). In the femur, cortical bone was evaluated in 20 slices (240 µm) in the femoral shaft with the first slice taken 60% distal to the top of the femoral head. Measurements in this region included cross-sectional volume (volume of cortical bone and bone marrow, mm^3^), cortical volume (mm^3^), marrow volume (mm^3^), cortical thickness (µm) and intracortical porosity. Cortical thickness was determined using the plate model and intracortical porosity was calculated as the difference between cortical bone volume at a threshold of 245 and a threshold of 0. Volume measurements in the cortical diaphysis were adjusted to a height of 1 mm. Measurements in the femur metaphysis were obtained in a total of 40 slices (480 µm) starting at 45 slices (540 µm) proximal to the growth plate and included bone volume fraction (bone volume/tissue volume, %), trabecular number (mm^−1^), trabecular thickness (µm), and trabecular spacing (µm). The same four parameters were also evaluated in the cancellous compartment (34±2 slices, 408±24 µm) in the femur epiphysis. The cancellous region of interest was delineated manually a few voxels away from the endocortical surface. For the tibia, cortical bone was evaluated in the tibia diaphysis (20 slices, 240 µm) starting 40 slices (480 µm) proximal to the tibiofibular junction and cancellous bone was evaluated in the proximal tibia metaphysis (40 slices, 480 µm) starting at 20 slices (240 µm) distal to the growth plate. Volume measurements in the cortical diaphysis in both femur and tibia were adjusted to a height of 1 mm. Cancellous bone was analyzed in both the femur metaphysis and epiphysis. Finally, cancellous bone volume fraction (%), trabecular number (mm^−1^), trabecular thickness (µm), and trabecular spacing (µm) were determined in the cancellous compartment of the vertebral body (secondary spongiosa between the cranial and caudal growth plates, 172±2 slices, 2064±24 µm).

### Histomorphometry

The histological methods used here have been previously described in detail [47]. In brief, 5^th^ lumbar vertebra were dehydrated in graded increases of ethanol and xylene and embedded in methyl methacrylate. Sections (4-µm thick) were cut with a vertical bed microtome (Leica/Jung 2165) and were stained according to the Von Kossa method with a tetrachrome counter stain (Polysciences, Warrington, PA, USA) for assessment of cell-based measurements. Osteoblast and osteoclast number and perimeter were measured in the entire cancellous compartment of the vertebral body. Osteoblast and osteoclast number was expressed per bone perimeter (#/mm) and osteoblast and osteoclast perimeter was expressed per total bone perimeter (%), bone area (mm/mm^2^) and tissue area (mm/mm^2^). Osteocyte number was also determined in the entire cancellous compartment of the vertebral body and expressed per total bone area (#/mm). Histomorphometric data were collected using the OsteoMeasure System (OsteoMetrics, Inc., Atlanta, GA, USA). All histomorphometric data are reported using standard 2-dimentional nomenclature [48].

### Biochemical markers of bone turnover

At the time of sacrifice, serum was collected via terminal bleeds from all mice. The serum levels of a bone formation marker, procollagen type 1 amino-terminal propeptide (P1NP), and a bone resorption marker, C-Telopeptide of Type I Collagen (CTX-1), were quantitated using ELISA kits from ImmunoDiagnostic Systems (Fountain Hills, AZ) as described by the manufacturer. All assays were performed in duplicate and averaged among the two treatment groups.

### Isolation of adherent marrow stromal cells and cortical shells of long bones

At the time of sacrifice, the right femur and tibia of 4-5 vehicle and endoxifen treated mice were collected and cleaned of all muscle tissue. Subsequently, the epiphyses were removed and bone marrow cells were flushed and collected in 1X PBS. Cells were plated in 6 well plates using α-MEM plus 10% fetal bovine serum (FBS) and a 1% antibiotic-antimycotic solution and cultured in a humidified 37°C incubator with 5% CO_2_ overnight. Twenty four hours after plating, non-adherent cells were removed and fresh media was added. Cells were allowed to proliferate for approximately 3 days and total RNA was then isolated using Trizol reagent (Invitrogen, Carlsbad, CA). Following removal of bone marrow from these femora and tibia, the cortical shells were immediately placed in Trizol reagent and homogenized for RNA extraction.

### Treatment of human fetal osteoblast cells

Human fetal osteoblasts cells expressing estrogen receptor α (FOB/ER9) were cultured as previously described [49]. FOB/ER9 cells were plated in 6 well tissue culture plates at approximately 50% confluence and subsequently treated with ethanol vehicle or endoxifen (100 nM or 1000 nM) in phenol-red free DMEM/F12 media containing 10% charcoal stripped FBS for 24 hours. Total RNA was collected using Trizol reagent.

### Isolation and differentiation of osteoclasts

Freshly isolated bone marrow cells were also collected from an additional 4-5 vehicle and endoxifen treated mice using the right femur and tibia of each animal as previously described [50]. Briefly, the epiphyses were removed and marrow cells were flushed and collected using 1X PBS. Marrow cells were cultured in α-MEM plus 10% fetal bovine serum (FBS) containing macrophage colony stimulating factor (MCSF) (25 ng/ml) for 24 hours. Non-adherent bone marrow cells were subsequently collected, seeded at an initial density of 4.5×10^5^ per well in 24-well plates, and cultured in the presence of receptor activator of nuclear factor kappa-B ligand (RANKL) (50 ng/ml) and MCSF (25 ng/ml). In parallel, cells were also cultured in the absence of MCSF to verify that there were no contaminating mesenchymal cells in the preparations. Osteoclast precursors were re-fed with the same media on day 3 and cells were fixed in 1% paraformaldehyde 24 hours later. To identify mature osteoclasts, cells were stained with Hoechst 33342 and for TRAP activity as previously described [51]. Total RNA was collected from an additional 4 wells of cells from each animal using Trizol reagent (Invitrogen) for examination of gene expression differences.

### RNA isolation and real-time PCR analysis

Total RNA was isolated from adherent marrow stromal cells and mature osteoclasts using Trizol reagent (Invitrogen) as specified by the manufacturer. RNA was quantitated using a NanoDrop 1000 spectrophotometer (Thermo Fisher Scientific, Wilmington, DE). Equal amounts of RNA originating from replicate mice in the vehicle and endoxifen treated groups were combined and cDNA was synthesized using the iScript™ cDNA Synthesis Kit (Bio-Rad, Hercules, CA). Real-time PCR was performed in triplicate as previously described [52] and values were normalized using β-tubulin as a control. All PCR primers were designed using Primer3 software (http://frodo.wi.mit.edu/primer3/) and were purchased from Integrated DNA Technologies (Coralville, IA). Primer sequences are listed in Table 1.

**Table 1:** Primer sets used in RT-PCR.

	Primer, 5′ - 3′	
Gene	Forward	Reverse
Runx2	GCCGGGAATGATGAGAACTA	GGTGAAACTCTTGCCTCGTC
Osterix	GGAGGTTTCACTCCATTCCA	TAGAAGGAGCAAGGGGACAGA
Alk. Phos.	TGAGCGACACGGACAAGA	GGCCTGGTAGTTGTTGTGAG
ERα	ATGACCCTTCACACCAAAGC	GCTTGCTGTTGTCCACGTAT
ERβ	CAGTCCATCCTACCCTTGGA	TGCTGCTGGGAACACTGTAG
NFATc1	TGATGGTGGCTTACCTTTCC	CTCTTCACAGTCGTGCGAAA
RANK	GCTGGGACCTGCAAATAAGT	GGGAAGCGTATACAGGGTCA
C-FMS	CTCAAAGGCTGTGGGTAAGC	GCCACTCCTGTGAGCTTAGG
CathK	CCAGTGGGAGCTATGGAAGA	AAGTGGTTCATGGCCAGTTC
OCIL	CCTGCACAGAGAGTCGTCAG	GGTACTGCTGATCCCGTTGT
MEPE	TGCTGCCCTCCTCAGAAATATC	GTTCGGCCCCAGTCACTAGA
PHEX	CCTTGGCTGAGACACAATGTTG	GCCTTCGGCTGACTGATTTCT
DMP1	F TGCTCTCCCAGTTGCCAGAT	AATCACCCGTCCTCTCTTCAGA
β Tubulin	CTGCTCATCAGCAAGATCAGAG	GCATTATAGGGCTCCACCACAG

### Statistical analyses

Prior to analysis, all data were reviewed by a dedicated statistician for normality and equality of variances using a Kolmogorov-Smirnov test for normality. Each of the parameters presented in this manuscript exhibited normal distributions and equal variances among animal groups. For these reasons, a 2-sided unpaired student's T-test was used to evaluate significant differences between vehicle and endoxifen treated mice. Statistical calculations were performed using Microsoft® Office Excel and all data are presented as the mean ± standard error. P-values <0.05 were considered to be statistically significant.

## Results

### DXA analysis of vehicle and endoxifen treated mice following ovariectomy

**Figure 1:**
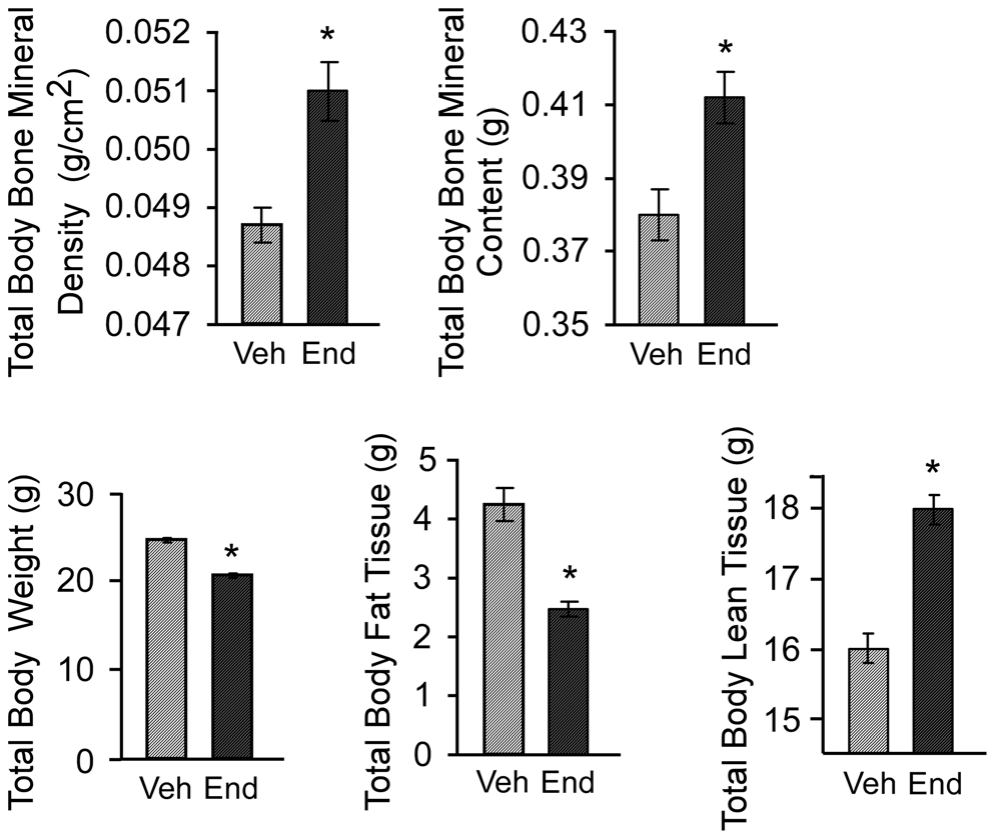
DXA analysis of ovariectomized mice following 45 days of vehicle (Veh) or endoxifen (End) treatment. Whole body bone mineral density (BMD), bone mineral content (BMC), total body weight, fat tissue mass and lean tissue mass are indicated. The mean ± SE are depicted. * denotes significance at P<0.05.

As a first step towards elucidating the potential effects of endoxifen on the skeleton, we utilized DXA analyses to measure total body bone mineral density and bone mineral content, as well as lean mass and fat mass following 45 days of vehicle or endoxifen treatment. Total body bone mineral density and content were significantly greater in the endoxifen treated mice relative to vehicle treated control animals (Figure 1). Interestingly, the weight of endoxifen treated animals was significantly lower than that of vehicle treated controls (Figure 1). This difference in body weight was accompanied by a significant decrease in total body fat mass, but a significant increase in total body lean mass, in endoxifen treated mice (Figure 1).

### pQCT analysis of the tibial metaphysis and diaphysis of vehicle and endoxifen treated mice following ovariectomy

**Figure 2:**
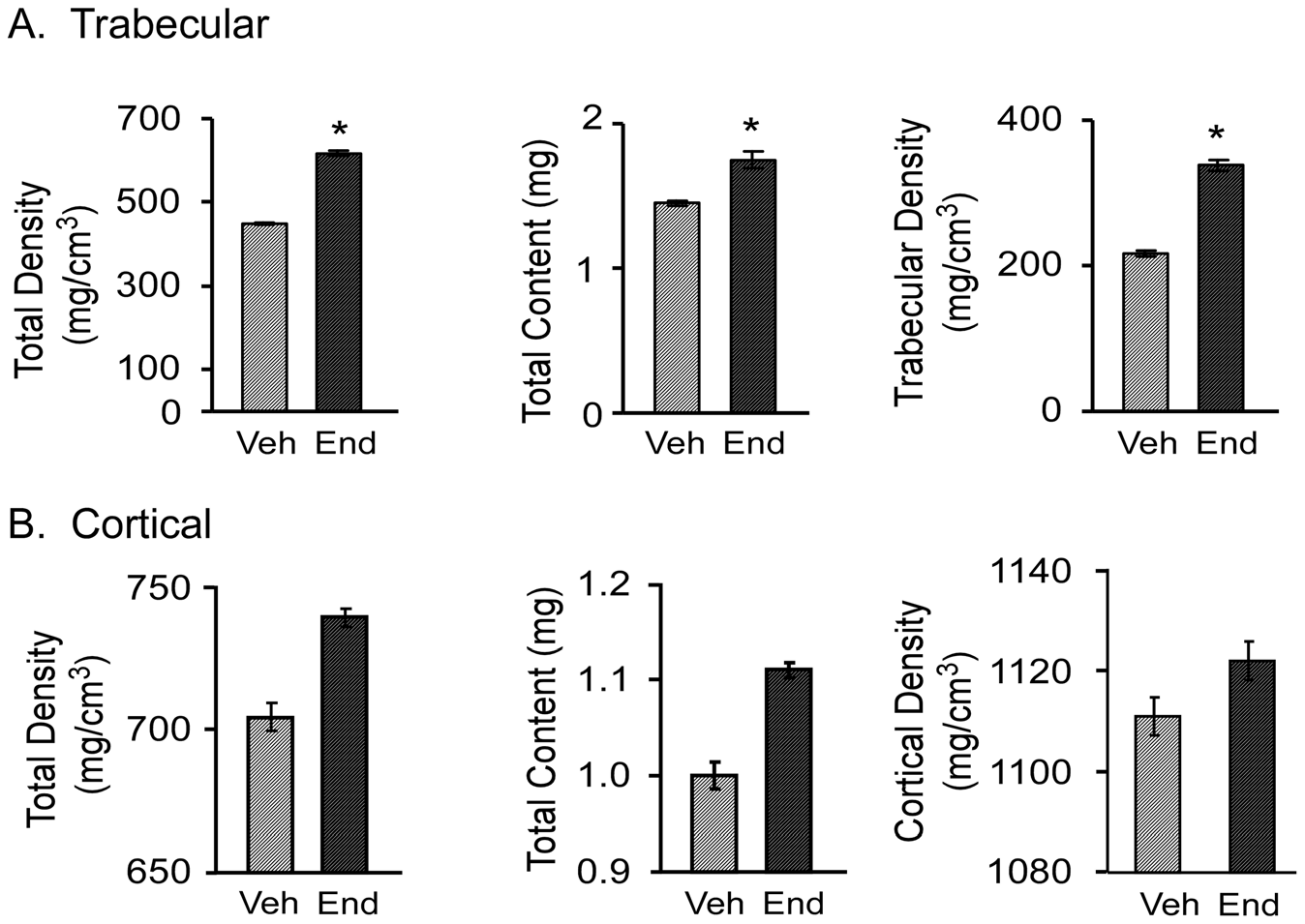
pQCT analysis of ovariectomized mice following 45 days of vehicle (Veh) or endoxifen (End) treatment. The tibial metaphysis (A) and tibial diaphysis (B) are indicated and include total bone mineral density, total bone mineral content, trabecular density, cortical density, cortical thickness, periosteal circumference, and endosteal circumference. The mean ± SE are depicted. * denotes significance at P<0.05.

To further analyze the effect of endoxifen on bone, we next examined a number of trabecular and cortical bone parameters in the tibial metaphysis and diaphysis respectively via pQCT (Figure 2). Endoxifen treated mice had significantly greater total bone mineral density and bone mineral content, as well as trabecular bone mineral density in the tibial metaphysis relative to vehicle treated animals (Figure 2A). In the tibial diaphysis, endoxifen treatment also resulted in greater total bone mineral density as well as cortical thickness compared to vehicle treatment (Figure 2B).

### Micro-CT analysis of the femur, tibia and vertebra

**Figure 3:**
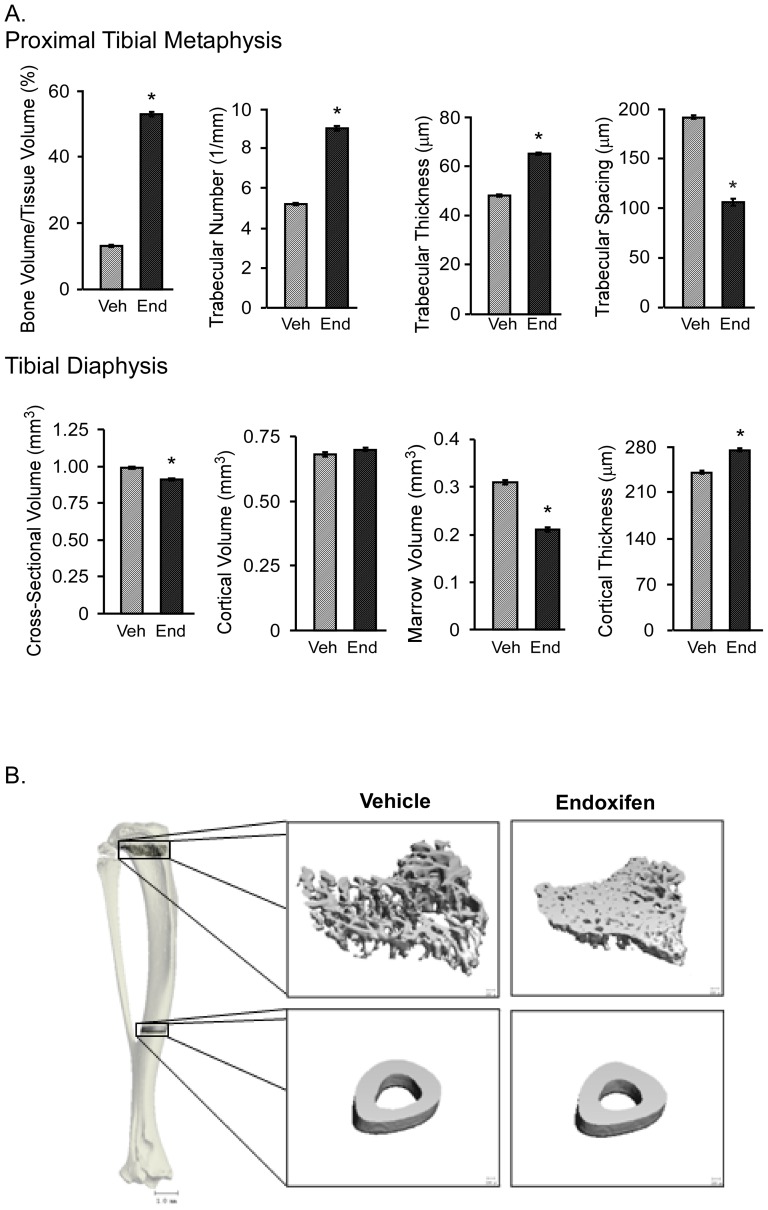
Micro-CT analysis of the tibia in ovariectomized mice following 45 days of vehicle (Veh) or endoxifen (End) treatment. A. The proximal tibial metaphysis and tibial diaphysis are indicated and include bone volume/tissue volume, trabecular number, trabecular thickness, trabecular spacing, cross-sectional volume, cortical volume, marrow volume and cortical thickness. The mean ± SE are depicted. * denotes significance at P<0.05. B. Representative micro-CT images of the cancellous bone and cortical bone from the tibia in a vehicle (control) and endoxifen treated animal are shown.

Following these studies, we analyzed the micro-architecture of long bones and vertebra from vehicle and endoxifen treated mice using micro-CT. With regard to the proximal tibial metaphysis, endoxifen treated animals exhibited greater bone volume to tissue volume ratio, trabecular number and trabecular thickness, and reduced trabecular spacing relative to vehicle treated mice (Figure 3A). In the tibial diaphysis, endoxifen treatment led to significantly reduced cross-sectional volume and marrow volume and a significant increase in cortical thickness (Figure 3A). No changes were observed with regard to cortical volume between the two groups of mice (Figure 3A). Representative micro-CT images of a vehicle and endoxifen treated mouse tibia are shown for these two sites in Figure 3B.

**Figure 4:**
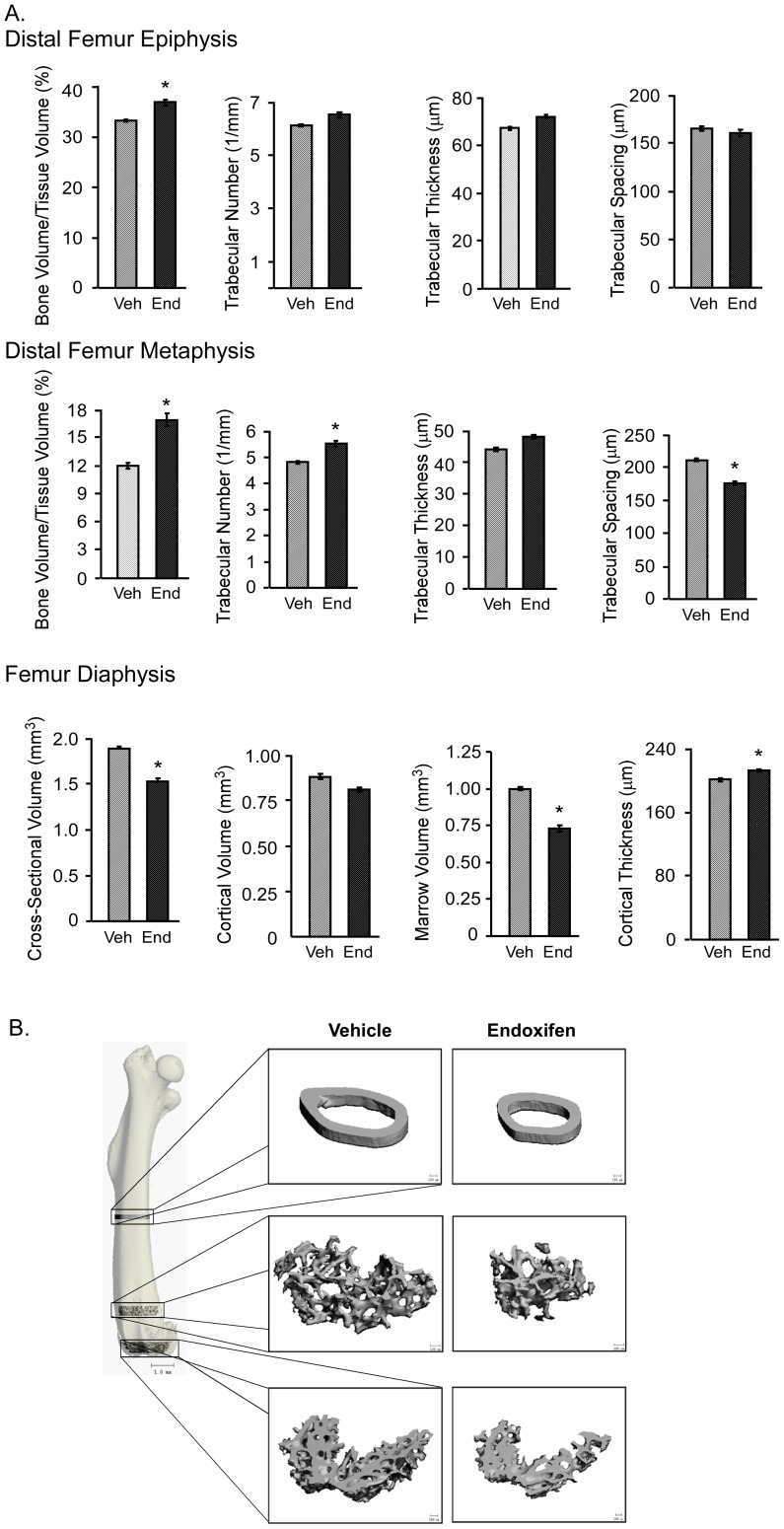
Micro-CT analysis of the femur in ovariectomized mice following 45 days of vehicle (Veh) or endoxifen (End) treatment. A. The distal femur epiphysis, metaphysis and midshaft diaphysis are indicated and include bone volume/tissue volume, trabecular number, trabecular thickness, trabecular spacing, cross-sectional volume, cortical volume, marrow volume and cortical thickness. The mean ± SE are depicted. * denotes significance at P<0.05. B. Representative micro-CT images of the cancellous bone and cortical bone from the femur in a vehicle (control) and endoxifen treated animal are shown.

At the femur epiphysis, the bone volume to tissue volume ratio was significantly greater in endoxifen treated mice relative to vehicle treated controls with no significant differences observed for trabecular number, thickness or spacing (Figure 4A). However, at the distal femur metaphysis, endoxifen treated mice exhibited significantly greater bone volume to tissue volume ratio and trabecular number with a concomitant decrease in trabecular spacing (Figure 4A). Similar to the tibia, cross-sectional volume, cortical volume and marrow volume were lower, while cortical thickness was higher, in endoxifen treated mice at the femur mid-shaft compared to vehicle treated animals (Figure 4A). Porosity was not significantly different in either the femoral or tibial cortical diaphysis (data not shown). Representative micro-CT images of a vehicle and endoxifen treated mouse femur are shown for these three sites in Figure 4B.

**Figure 5:**
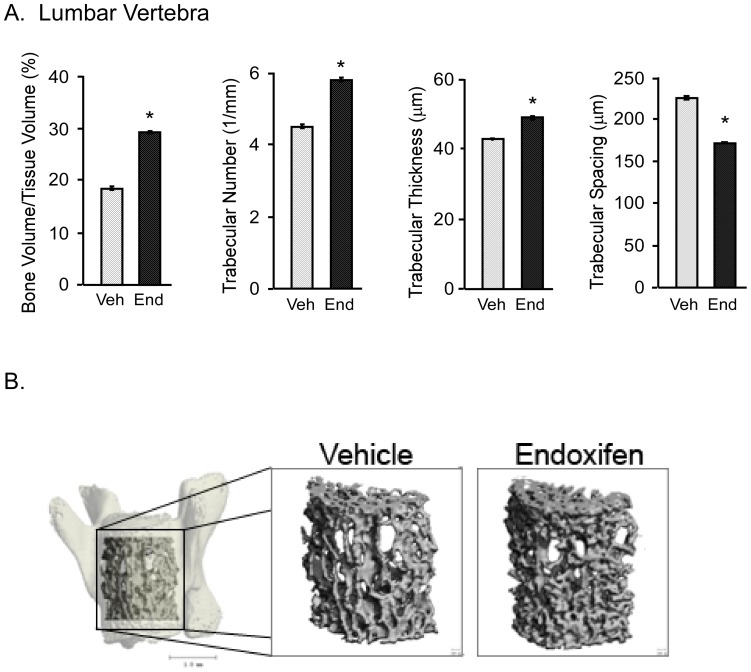
Micro-CT analysis of the 5^th^ lumbar vertebrae in ovariectomized mice following 45 days of vehicle (Veh) or endoxifen (End) treatment. A. Bone volume/tissue volume, trabecular number, trabecular thickness and trabecular spacing are indicated. The mean ± SE are depicted. * denotes significance at P<0.05. B. Representative micro-CT images of the cancellous bone from the 5^th^ lumbar vertebra in a vehicle (control) and endoxifen treated animal are shown.

In the 5^th^ lumbar vertebra, the bone volume to tissue volume ratio was significantly greater in endoxifen treated animals relative to vehicle treated controls (Figure 5A). Additionally, significantly higher trabecular number and trabecular thickness, as well as lower trabecular spacing were observed at this bone site following endoxifen treatment (Figure 5A). Representative micro-CT images of the 5^th^ lumbar vertebra from a vehicle and endoxifen treated mouse are shown in Figure 5B.

### Histomorphometric analysis of the 5^th^ lumbar vertebra

Based on the dramatic changes observed in cancellous bone following endoxifen treatment, we next performed histomorphometry on the 5^th^ lumbar vertebra in order to assess potential endoxifen mediated changes at the cellular level. Interestingly, endoxifen treatment led to significantly higher osteoblast perimeter/tissue area (Table 2) but had no effect on osteoblast perimeter/bone perimeter, osteoblast perimeter/bone area or osteoblasts/bone perimeter. With regard to osteoclasts, a significant increase in osteoclast perimeter/tissue area was also observed with no changes in osteoclast perimeter/bone perimeter osteoclast perimeter/bone area or osteoclasts/bone perimeter in endoxifen treated animals relative to vehicle treated controls (Table 2). The number of osteocytes per bone area was increased in endoxifen treated animals although this parameter did not reach statistical significance (p = 0.06).

**Table 2:** Histomorphometric Analysis of the 5th Lumbar Vertebrae.

Endpoint	Vehicle (n = 9)	Endoxifen (n = 6)
Osteoblast Perimeter/Bone Perimeter (%)	12.2±1.9	15.9±2.3
Osteoblast Perimeter/Bone Area (mm/mm^2^)	5.9±1.1	7.4±1.2
Osteoblast Perimeter/Tissue Area (mm/mm^2^)	0.9±0.1	1.6±0.3*
Osteoblasts/Bone Perimeter (#/mm)	11.89±2.11	15.48±2.41
Osteoclast Perimeter/Bone Perimeter (%)	2.7±0.7	2.7±0.3
Osteoclast Perimeter/Bone Area (mm/mm^2^)	1.1±0.2	1.3±0.2
Osteoclast Perimeter/Tissue Area (mm/mm^2^)	0.2±0.0	0.3±0.0*
Osteoclasts/Bone Perimeter (#/mm)	0.69±0.14	0.83±0.05
Osteocytes/Bone Area (#/mm^2^)	554±26	639±31
N	9	6

Data are mean ± SE. * P<0.05.

### Bone formation and resorption markers

**Figure 6:**
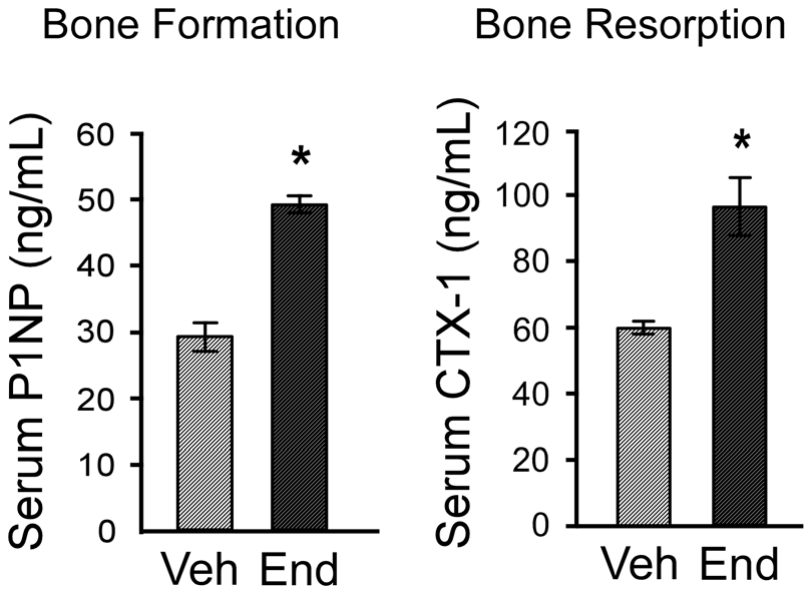
Serum levels of bone turnover markers in vehicle and endoxifen treated mice. ELISAs were used to determine the levels of the bone formation marker, P1NP, and the bone resorption marker, CTX-1, following 45 days of vehicle (Veh) and endoxifen (End) treatment. The mean ± SE are depicted. * denotes significance at P<0.05.

Since endoxifen treatment appeared to increase the numbers of both osteoblasts and osteoclasts as determined by histomorphometric analysis of the 5^th^ lumbar vertebra, it was of interest to examine potential changes in the serum levels of biochemical markers of bone turnover. Specifically, P1NP, an indicator of bone formation, and CTX-1, a marker of bone resorption, were determined by ELISA. The serum levels of both of these markers were significantly elevated in endoxifen treated mice relative to vehicle treated controls (Figure 6).

### Real-time PCR analysis of bone marker genes in adherent marrow stromal cells, FOB/ER9 cells and cortical shells of long bones

**Figure 7:**
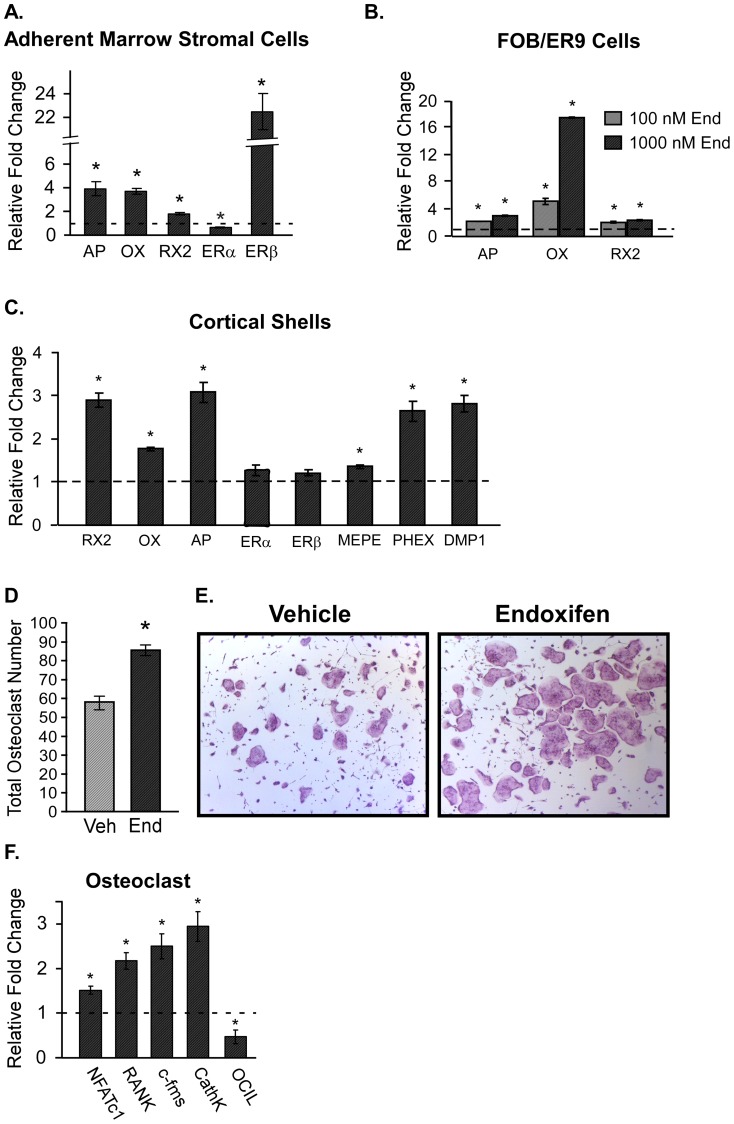
Cellular responses to vehicle and endoxifen treatment. A. Real-time PCR analysis of alkaline phosphatase (AP), osterix (OX), Runx2 (RX2), estrogen receptor α (ERα) and estrogen receptor β (ERβ) in adherent marrow stromal cells derived from endoxifen treated mice relative to vehicle treated control animals. B. Real-time PCR analysis of AP, OX, and RX2 following 24 hour treatment of human fetal osteoblast cells expressing ERα (FOB/ER9) with 100 nM or 1000 nM levels of endoxifen. C. Real-time PCR analysis of AP, OX, RX2, ERα, ERβ, matrix extracellular phosphoglycoprotein (MEPE), phosphate-regulated neutral endopeptidase (PHEX) and dentin matrix acidic phosphoprotein1 (DMP1) in cortical shells isolated from endoxifen treated mice relative to vehicle treated control animals. D. Quantification of TRAP positive osteoclasts (OC) after MCSF and RANKL treatment of non-adherent bone marrow cells isolated from vehicle (Veh) or endoxifen (End) treated mice. E. A representative image of differentiated osteoclasts from vehicle and endoxifen treated mice. F. RT-PCR analysis of the osteoclast marker genes NFATc1, RANK, c-Fms and CathK, as well as the inhibitory OCIL gene, in mature osteoclasts derived from endoxifen treated animals relative to vehicle treated controls. The mean ± SE are depicted. * denotes significance at P<0.05.

To further examine the effects of endoxifen treatment at the molecular level, we analyzed the expression levels of a number of important osteoblast marker genes, as well as ERα and ERβ, in osteoblast precursor cells isolated from vehicle and endoxifen treated mice. As shown in Figure 7A, significant increases in the expression levels of alkaline phosphatase (AP), osterix (OX) and Runx2 (RX2) were observed. Additionally ERα was significantly repressed and ERβ was significantly induced following endoxifen treatment (Figure 7A). To determine if these effects of endoxifen were also elicited in a more mature osteoblast cell, human FOB/ER9 cells were analyzed following 24 hours of endoxifen treatment *in vitro*. Interestingly, these same osteoblast marker genes were significantly induced by two different doses of endoxifen relative to vehicle control treated cells (Figure 7B). These effects were not observed in the parental hFOB cell line which does not express either ERα or ERβ demonstrating that these effects of endoxifen are elicited through the actions of the estrogen receptor (data not shown). To further evaluate the effects of endoxifen treatment on mature osteoblasts/terminally differentiated osteocytes, the expression of these genes, as well as classic osteocyte marker genes, were evaluated in the cortical shells of long bones isolated from endoxifen and vehicle treated animals. As shown in Figure 7C, AP, OX and RX2 were significantly induced in endoxifen treated animals. Similarly, the osteocyte marker genes, matrix extracellular phosphoglycoprotein (MEPE), phosphate-regulated neutral endopeptidase (PHEX) and dentin matrix acidic phosphoprotein 1 (DMP1), were elevated in endoxifen treated mice compared to vehicle treated controls (Figure 7C). In contrast to the adherent marrow stromal cells, ERα and ERβ expression levels were minimally affected in the cortical shells of long bones (Figure 7C).

### Osteoclast differentiation and gene expression

In addition to analyzing gene expression differences in bone marrow stromal cells, we also examined the effects of endoxifen on the osteoclast precursor cell population within the bone marrow. As shown in Figure 7D, there was a significant increase in the number of TRAP positive osteoclasts following MCSF and RANKL treatment of non-adherent bone marrow cells isolated from endoxifen treated mice relative to vehicle treated control animals. Also of note was the observation that osteoclasts derived from endoxifen treated mice were larger in size as depicted in the representative image shown in Figure 7E. Increased expression of the osteoclast marker genes NFATc1, RANK, c-fms and cathepsin-K, as well as decreased expression of the inhibitory OCIL gene, were detected in mature osteoclasts derived from endoxifen treated animals relative to vehicle treated controls (Figure 7F).

## Discussion

Our past studies have provided evidence that endoxifen is a potent active metabolite of tamoxifen which appears to substantially contribute to the anti-cancer effects of this drug. In part due to these past studies, the NCI has developed a novel formulation of endoxifen which is now being used in phase I clinical trials at the Mayo Clinic and the NCI for treatment of endocrine sensitive breast cancer (NCT01327781 and NCT01273168). Since tamoxifen and other drugs which are classified as SERMs have known effects on the skeleton, we sought to characterize the effects of endoxifen on bone using a mouse model system. As mentioned previously, the potential effects of endoxifen on the skeleton cannot be extrapolated from past rodent studies examining the actions of tamoxifen on bone since the concentrations of endoxifen have been shown to be extremely low in tamoxifen treated murine models [44], [45]. Here, we have demonstrated that an anti-cancer dose of endoxifen enhances bone mineral density and content throughout the skeleton in an ovariectomized mouse model. The largest percent changes were generally observed in cancellous bone with more modest effects on cortical bone at the dose and time point analyzed. At the cellular level, endoxifen treatment led to tissue level increases in osteoblast and osteoclast perimeter and corresponding increases in serum concentrations of biochemical markers of bone formation (P1NP) and resorption (CTX-1) suggesting that endoxifen may increase bone turnover in the mouse. The fact that endoxifen treated animals appear to have a high bone mass phenotype in the presence of higher rates of bone turnover suggests that endoxifen may also enhance coupling between osteoblasts and osteoclasts, a potential effect that warrants further exploration.

There is a substantial amount of data demonstrating that a number of SERMs can protect against bone loss following estrogen depletion in various animal model systems and act to preserve bone mass in post-menopausal women (reviewed in: [53], [54], [55]). Tamoxifen and raloxifene are the two most well studied SERMs with regard to their effects on the skeleton. In ovariectomized mice, treatment with tamoxifen has been shown to result in dramatic increases in a number of cancellous bone parameters as determined by micro-CT analysis [56]. However, no changes in cortical bone were observed in this past study [56]. Similarly, raloxifene enhances cancellous bone in the distal femur of ovariectomized mice with little to no changes observed in cortical bone [57]. These data display similarities with the endoxifen effects presented here, demonstrating that endoxifen exposure results in significant increases in multiple cancellous bone parameters throughout the mouse skeleton as determined by DXA, pQCT and micro-CT. In contrast however, our results also indicate that high concentrations of endoxifen enhance cortical bone thickness in ovariectomized mice. The effects of endoxifen on cortical bone observed here, that have not been reported in previous studies of tamoxifen or raloxifene, could be reflective of dosage differences and/or the age of the animals used in the experiments. Previous studies from our laboratory have also demonstrated that the molecular mechanisms of endoxifen action are dramatically different in breast cancer cells compared to other SERMs [43]. Based on these data, it is not unrealistic to assume that the molecular mechanisms of endoxifen action may also differ substantially in bone, particularly when administered at anti-cancer doses as has been done in the present study.

At the cellular level, SERMs are considered to be primarily anti-resorptive therapies since they repress osteoclast differentiation and activity with lesser effects on osteoblasts. Tamoxifen and raloxifene have been shown to reduce osteoclast differentiation *in vitro* [58], [59], [60], [61] and repress histomorphometric indices of bone resorption in ovariectomized rats [62], [63], [64], [65]. In contrast to these studies, our histomorphometric analyses of the 5^th^ lumbar vertebra revealed tissue level increases in osteoclast-lined bone perimeter. This result is consistent with our observation of increased serum levels of CTX-1, a biochemical marker of bone resorption, in endoxifen treated mice.

*In vitro* studies have also demonstrated that tamoxifen and raloxifene can induce the expression of Runx2 in osteoblasts [66]. Others have also shown that raloxifene can stimulate osteoblast proliferation and induce expression of osteoblast marker genes such as Runx2 and collagen type 1 [60], TGFβ3 [67] and BMP4 [68]. Additionally, tamoxifen and raloxifene can stimulate osteoblastic differentiation of mouse bone marrow stromal cells *in vitro* [69]. However, raloxifene treatment of rats has been shown to repress osteoblast activity as indicated by decreases in osteoblast perimeter, calcein-labeled perimeter, mineral apposition rate and bone formation rates [64]. In this study, we have provided evidence that endoxifen exposure increases osteoblast perimeter per tissue area and results in increased serum P1NP levels. Furthermore, our studies have revealed that endoxifen also induces the expression of classic osteoblast marker genes both *in vivo*, using adherent marrow stromal cells isolated from endoxifen treated mice, as well as *in vitro*, using the human FOB/ER9 cell line. Additionally, our data suggest that endoxifen's effects on the mouse skeleton may also occur through the actions of osteocytes due to the fact that increased expression of bone marker and osteocyte marker genes were observed in the cortical shells of long bones isolated from endoxifen treated mice. These gene expression studies are consistent with our observation that the numbers of osteocytes embedded within the bone are increased following endoxifen exposure.

SERMs are primarily known to function by binding to ERα and ERβ to elicit transcriptional responses. Both ERα and ERβ are expressed in bone, however; their relative expression levels appear to differ based on the bone compartment and cell type. Specifically, ERα is primarily expressed in osteoblasts and osteocytes located in cortical bone while ERβ is more highly expressed in these two cell types in cancellous bone [70]. Additionally, both ERs were shown to be expressed in osteoclasts in both cortical and cancellous compartments [70]. It is interesting to note that the expression levels of ERβ, but not ERα, were significantly increased in bone marrow stromal cells isolated from endoxifen treated mice relative to placebo treated animals suggesting that ERβ may be a critical mediator of endoxifen's effects on this particular cell population and may contribute to some of the increases in osteoblast numbers and bone mass reported in this study.

In summary, these studies are the first to examine the *in vivo* impact of endoxifen on bone and have revealed that endoxifen increases cancellous as well as cortical bone mass in ovariectomized mice. The mechanisms by which endoxifen elicits these effects on bone appear to occur, at least in part, through regulating the functions of the three major bone cell types. Our data suggest that this novel breast cancer therapy may elicit beneficial effects on bone in post-menopausal breast cancer patients, an effect that is being evaluated in the ongoing endoxifen clinical trials.
